# Rewarding-unrewarding prediction signals under a bivalent context in the primate lateral hypothalamus

**DOI:** 10.1038/s41598-023-33026-0

**Published:** 2023-04-12

**Authors:** Atsushi Noritake, Kae Nakamura

**Affiliations:** 1 Division of Behavioral Development, Department of System Neuroscience, National Institute for Physiological Sciences, National Institutes of Natural Sciences, Okazaki, 444-8585 Japan; 2 Department of Physiological Sciences, School of Life Science, The Graduate University for Advanced Studies (SOKENDAI), Hayama, 240-0193 Japan; 3 Department of Physiology, Kansai Medical University, 2-5-1, Shinmachi, Hirakata, Osaka 573-1010 Japan

**Keywords:** Motivation, Reward

## Abstract

Animals can expect rewards under equivocal situations. The lateral hypothalamus (LH) is thought to process motivational information by producing valence signals of reward and punishment. Despite rich studies using rodents and non-human primates, these signals have been assessed separately in appetitive and aversive contexts; therefore, it remains unclear what information the LH encodes in equivocal situations. To address this issue, macaque monkeys were conditioned under a bivalent context in which reward and punishment were probabilistically delivered, in addition to appetitive and aversive contexts. The monkeys increased approaching behavior similarly in the bivalent and appetitive contexts as the reward probability increased. They increased avoiding behavior under the bivalent and aversive contexts as the punishment probability increased, but the mean frequency was lower under the bivalent context than under the aversive context. The population activity correlated with these mean behaviors. Moreover, the LH produced fine prediction signals of reward expectation, uncertainty, and predictability consistently in the bivalent and appetitive contexts by recruiting context-independent and context-dependent subpopulations of neurons, while it less produced punishment signals in the aversive and bivalent contexts. Further, neural ensembles encoded context information and “rewarding-unrewarding” and “reward-punishment” valence. These signals may motivate individuals robustly in equivocal environments.

## Introduction

Animals, including humans and non-human primates, may expect and evaluate rewards even under situations in which rewards and punishments can both be outcomes. As such situations are bivalent and equivocally interpretable, they may induce different approaching and avoiding behaviors. When animals are concerned about negative outcomes, they may devalue bivalent situations compared to those in which rewards alone are obtained. This bias results in a reduction in the frequency of approaching behavior. Conversely, when animals focus on positive outcomes, they may overestimate the value of bivalent situations compared to those in which punishments alone are obtained, thereby reducing in the frequency of avoiding behavior. Alternatively, they may integrate information on both outcomes and show compromised behavior. These differences in perspective reflect the processing of rewarding and punishing information, which motivates animals under such equivocal situations.

The lateral hypothalamus (LH) is thought to function as a node in the processing of information related to approaching motivation for food and water and avoiding motivation such as escaping predators, in addition to arousal and energy homeostasis^1–8^. Anatomically, the LH has direct and indirect reciprocal connections with regions essential for reward- and punishment-information processing such as the ventral tegmental area^5,9,10^, nucleus accumbens shell^11,12^, ventral pallidum^11,13,14^, amygdala^15,16^, lateral habenula^17–19^, and periaqueductal gray matter^20,21^, and the forebrain system processing arousal and attention signals by cholinergic modulation such as the septum^22–24^ and locus coeruleus^9,25,26^. Therefore, the LH is one of suitable neural substrates underlying behaviors in such bivalent situations.

Indeed, rodent studies suggest that the LH encodes reward-punishment valence signals^27^. Studies using non-human primates also demonstrated that LH neurons carry signals related to reward and punishment prediction including expectation, appreciation, and uncertainty and respond to punishing events^28–30^. Despite such a rich literature, previous studies assessed neuronal activity separately in reward and aversive contexts in which rewards alone (appetitive block) or punishments alone (aversive block) were used. It remains unclear how behaviors and neuronal activity in the LH are influenced in bivalent situations when rewards and punishments may occur with equal frequency in the same context (bivalent block).

To address these issues, we introduced macaque monkeys to a bivalent block under a Pavlovian trace procedure in which rewards and punishments could occur with equal frequency in the same block with probability, in addition to appetitive and aversive blocks in which rewards and punishments alone occurred, respectively.

## Results

### Conditioning

**Figure 1:**
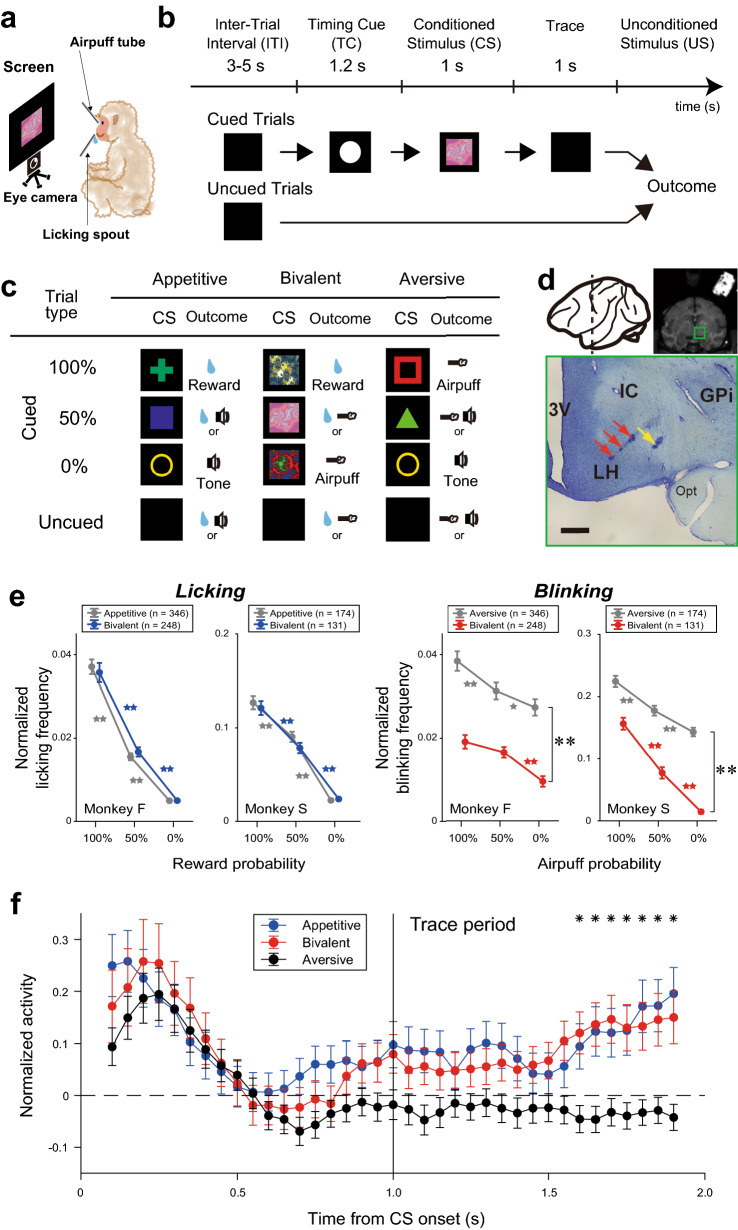
Pavlovian trace conditioning procedure and behaviors. (**a**) Experimental setting. Licking spout and airpuff tube were set toward the mouth and right lateral canthus, respectively. (**b**) Sequence of the Pavlovian trace-conditioning procedure. (**c**) Conditioned stimulus (CS) and outcome matrix in the appetitive (*left*), bivalent (*center*), and aversive (*right*) blocks. (**d**) Recording sites. Schema of the left hemisphere from the left view (*upper left*), coronal section of a magnetic resonance image (*upper right*; monkey S), and Nissl-stained section (*bottom*; scale bar, 1 mm; monkey S) for the area indicated by the green rectangle. Red arrows, electrolytic microlesions made in the lateral hypothalamus (LH). The distance between microlesions is 500 µm. Yellow arrow, electrolytic microlesion at 1-mm lateral to the grid coordinate made at the entry point into the LH. GPi, internal globus pallidus; IC, internal capsule; OPT, optic tract. (**e**) Licking behavior in the bivalent and appetitive blocks (*left*) and blinking behavior in the bivalent and aversive blocks (*right*). Means ± standard error of the mean of the normalized frequency during the last 250 ms of the trace period in monkeys F and S. *p < 0.05/3; **p < 0.01/3, Wilcoxon signed-rank test with Bonferroni’s correction. **Statistically significant differences in blinking frequency between the aversive and bivalent blocks (*F*_[1,1778]_ = 63.4, *p* = 2.94^–15^, two-way analysis of variance). (**f**) Population activity comparison among the three blocks during the conditioned stimulus (CS) and trace periods. Each dot depicts the mean normalized activity of the 244 neurons in a time bin in the appetitive (*blue*), bivalent (*red*), and aversive (*black*) blocks. Each bin has a 200-ms duration and moves in 50-ms steps from CS onset to the end of trace period. Data were plotted at the half time of the duration. Significant lower activity in the aversive blocks than that in the appetitive and bivalent blocks was represented by asterisks (*p* < 0.05, one-way ANOVA and *post-hoc* Tukey–Kramer test).

Two macaque monkeys were conditioned under appetitive, aversive, and bivalent blocks (Fig. 1a–c). The procedures in the appetitive and aversive blocks were described in detail elsewhere^28^. A bivalent block consisted of cued and uncued trials, as did the appetitive and aversive blocks. In the cued trials (Fig. 1b, cued trials), a water or juice reward was delivered with a 100%, 50%, or 0% probability, each of which was associated with one of three different conditioned stimuli (CSs) (Fig. 1c, *center*). When the reward was not received on the 50% and 0% trials, an airpuff was delivered instead. This differed from the delivery of a tone used in the appetitive and aversive blocks when an unconditioned stimulus (US; a reward or an airpuff) was not delivered. Accordingly, the probabilities in the bivalent blocks indicate the frequencies of reward, not punishment. In the uncued trials, a reward or an airpuff was delivered at the time corresponding to that of outcome delivery in the cued trials to manipulate the degree of reward and punishment predictability (Fig. 1b, uncued trials). These cued and uncued trials were presented pseudorandomly in a block (Methods). The appetitive and aversive blocks were always conducted before the bivalent blocks so that the experience of these blocks could serve as the basis for the valuation of the rewards and punishments in the bivalent blocks.

### Behavioral valuation of CSs

We assessed anticipatory licking and blinking frequency during the last 250 ms of the trace period as positive and negative evaluations, respectively, of the CSs. In the bivalent blocks, anticipatory licking frequency increased as the reward CS probability increased (Fig. 1e, *left, blue*), similarly to that in the appetitive blocks (Fig. 1e, *left, gray*). Anticipatory blinking frequency increased in the bivalent blocks as the punishment probability increased (Fig. 1e, *right, red*), similarly to that in the aversive blocks (Fig. 1e, *right, gray*); however, mean blinking frequency was significantly lower. These results suggest that in the bivalent blocks, predicting an airpuff did not influence anticipatory approaching behavior (i.e., anticipatory licking) for appetitive CS valuation, whereas predicting a reward reduced avoidance behavior (i.e., anticipatory blinking) for aversive CS valuation. Thus, the bivalent blocks did not alter reward valuation, but lowered punishment estimation.

### Activity modulation in the LH among the three blocks

A total of 244 neurons (monkey F, *n* = 127; monkey S, *n* = 117) in the LH (Fig. 1d) were tested in the bivalent blocks, which were the task-related neurons (*n* = 308) analyzed in a previous study^28^ (Methods). We first analyzed activity modulation at the population level among the three blocks. Activity peaked after CS onset in the three blocks similarly (one-way analysis of variance [ANOVA], *p* < 0.05). According to the reward-punishment valence coding hypothesis^31^, neurons respond to both reward and punishment, and these results support the notion that the LH encodes reward-punishment valence. Notably, activity modulation during the last 400 ms of the trace period was significantly higher in the bivalent blocks than in the aversive blocks, but not in the appetitive blocks (ANOVA and *post-hoc* Tukey–Kramer test, *p* < 0.05; Fig. 1f). This activity might function as positive motivation to reduce blinking behavior in the bivalent blocks compared to the aversive blocks and retain similar licking behavior between the bivalent and appetitive blocks. These results suggest that the LH also encode rewarding-unrewarding valence with a different time course at the population level. However, this activity did not explain the graded behavior that was depending on the associated outcome probabilities.

### Graded responses to CS values with bidirectional responses

**Figure 2:**
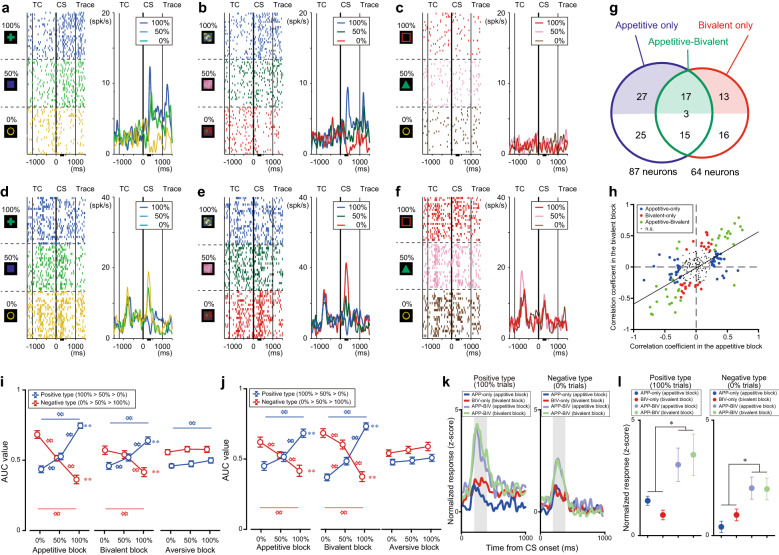
Conditioned stimulus (CS) value-coding neurons in the lateral hypothalamus. (**a–f**) Representative responses of a positive CS value-coding neuron in the appetitive (**a**), bivalent (**b**), and aversive (**c**) blocks and a negative type in the appetitive (**d**), bivalent (**e**), and aversive (**f**) blocks in the rasters (*left*) and histograms (*right*). TC, timing cue. Horizontal black bars indicate the analysis time window. (**g**) Number of CS value-coding neurons in the appetitive and bivalent blocks. The labels indicate the CS value-coding neurons with significantly correlated activity according to reward probability only in the appetitive blocks (blue), only in the bivalent blocks (red), and in both blocks (green), respectively. Colored and white areas depict positive and negative types, respectively. Digit on the border between the colored and white areas is the number of neurons with different correlation signs between the blocks. (**h**) Consistent sensitivity of CS values between the appetitive and bivalent blocks (*p* = 1.86^–20^, *ρ* = 0.55, Spearman’s correlation test). Each dot represents a single lateral hypothalamus neuron. Dot colors depict neuron types in g. (**i,j**) Preferred responses of CS value-coding neurons with significant CS value-dependent activity in the appetitive (**i**) or bivalent (**j**) blocks. The responses were indexed by the area under the curve (AUC) value during the test window (201–400 ms after CS onset) relative to the responses for the last 500 ms before timing cue (TC) onset (**p < 0.01/3, Wilcoxon signed-rank test with Bonferroni’s correction; **p < 0.01, Spearman’s correlation test). (**k–l**) Larger response modulation of the appetitive-bivalent (APP-BIV) neurons than that of the appetitive-only (APP-only) and bivalent-only (BIV-only) neurons (**k**) and mean values during 201–400 ms after CS onset (gray areas in k) for the positive (*left*) and negative (*right*) types (**l**). Normalized activity (*z*-score) was computed by using the activity during the last 500 ms before TC onset. The activity in the 100% trials was compared between the blocks for the positive types (*left*), while that in the 0% trials was compared for the negative types (*right*). *p < 0.05, one-way ANOVA and *post-hoc* Fisher’s least significant difference test.

To capture the graded responses to CS values associated with reward probability in parallel with the graded approaching behaviors, we applied correlation testing between CS values and the mean CS activity (201–400 ms after CS onset). In the appetitive blocks, 36% (87/244) of neurons exhibited responses that were correlated with the CS values (“CS value-coding” neurons). Among them, 48% (42/87) had a positive correlation (“positive type;” representative example, Fig. 2a; population, Fig. S1a,b), while the remaining 52% (45/87) had a negative correlation (“negative type;” representative example, Fig. 2d; population, Fig. S1d,e). The activity of these neurons did not clearly differentiate the CS values predictive for airpuffs (punishment CS values) (representative examples, Fig. 2c,f; population, Fig. S1c,f). In the bivalent blocks, 26% (64/244) of the same population were classified as CS value-coding neurons. The proportion of positive and negative types was similar to that in the appetitive blocks (positive type: 32/64; negative type, 32/64). A subset of the tested neurons exhibited graded activity consistently between the bivalent and appetitive blocks (“appetitive-bivalent” neurons, *n* = 35; representative examples: Fig. 2a,b and d,e; population, Fig. 2g, *green*); however, different subsets exhibited context-dependent graded responses either in the appetitive (“appetitive-only” neurons, *n* = 52; Fig. 2g, *blue*) blocks or bivalent (“bivalent-only” neurons, *n* = 29; Fig. 2g, *red*) blocks.

To examine the relationship of response sensitivity to CS values between the appetitive and bivalent blocks, we plotted the correlation coefficients of individual neurons between the CS response and CS values in the bivalent blocks against those in the appetitive blocks (Fig. 2h). There was a significantly positive correlation between the blocks (*p* < 0.01, Spearman’s correlation test), indicating that the more discriminative the neurons were for CS values in the appetitive blocks, the more discriminative they were in the bivalent blocks. Moreover, the neurons with correlations in the appetitive blocks (appetitive-only and appetitive-bivalent types) differentiated the CS values in the appetitive blocks more than those in the bivalent blocks (Fig. 2i, *left and center*). In contrast, the neurons with correlations in the bivalent blocks (bivalent-only and appetitive-bivalent types) differentiated the CS values in the bivalent blocks more than those in the appetitive blocks (Fig. 2j, *left and center*). These neurons did not differentiate punishment CS values (Fig. 2i,j, *right*). Further, the neurons with a significant correlation in the appetitive and bivalent blocks (appetitive-bivalent neurons) had a significantly larger response than those with a significant correlation in either block (appetitive-only neurons and bivalent-only neurons) in the 100% trials for the positive-type neurons (Fig. 2k,l, *left*) and the 0% trials for the negative-type neurons (Fig. 2k,l, *right*). Thus, the LH produced similar bidirectional responses to reward-predicting cues in the bivalent and appetitive blocks. These consistent responses, albeit with different outcome ranges, were accomplished by recruiting shared and context-dependent subpopulations of neurons.

We also quantified how many of the neurons that could be classified by CS value-dependent responses in the aversive blocks exhibited similar correlated responses in the bivalent blocks. A small but notable number of neurons exhibited significant response modulation that was dependent on punishment CS values (27/244; positive type: 6/27; negative type: 21/27). In addition to the reward CS-value coding neurons, this activity supports the notion that the LH encodes reward-punishment valence. Since the bivalent blocks were equivocally interpretable in the opposite manner such that the probability of an airpuff being delivered increased as the reward probability decreased, the responses of the punishment CS value-coding neurons might be similar to those of the negative-type neurons in the bivalent blocks. Approximately 52% of neurons (14/27) showed a significant correlation in the aversive and bivalent blocks, and 51% (8/14) of them encoded graded punishment CS values consistently between the aversive and bivalent blocks (positive type: *n* = 2; negative type: *n* = 6). This was in contrast with the observation that most of the neurons (Fig. 2h; 32/35) consistently encoded the graded reward CS values between the appetitive and bivalent blocks. In addition, there was no significant correlation between the bivalent and aversive blocks at the population level (*p* = 0.87, Spearman’s correlation test). These results suggest that the LH predominantly encodes the opposing rewarding-unrewarding valence of the CS values, in addition to the reward-punishment valence, at the cellular and population levels after CS onset.

### Graded responses to reward predictability with bidirectional responses

**Figure 3:**
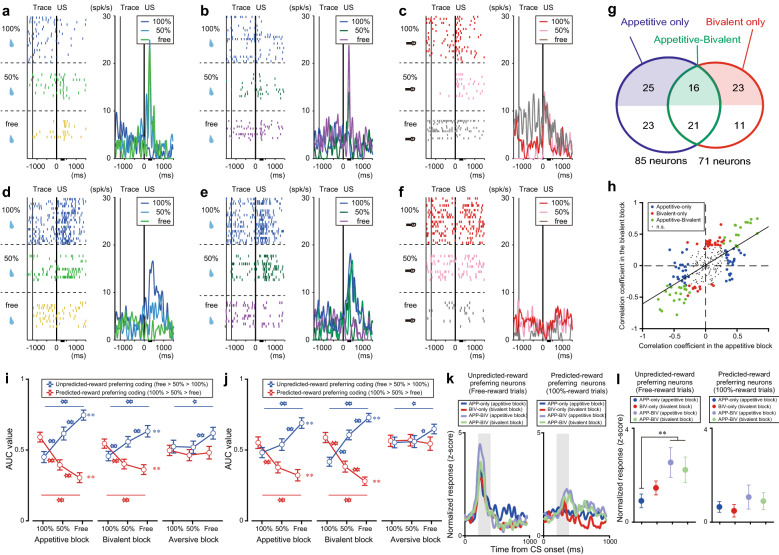
Reward predictability-coding neurons in the lateral hypothalamus. (**a–f**) Representative responses of an unpredicted reward-preferring neuron in the appetitive (**a**), bivalent (**b**), and aversive (**c**) blocks in the rasters (*left*) and histograms (*right*) and a predicted reward-preferring neuron in the appetitive (**d**), bivalent (**e**), and aversive (**f**) blocks. US, unconditioned stimulus. (**g**) Number of reward predictability-coding neurons in the appetitive and bivalent blocks. The labels indicate the reward predictability-coding types (appetitive-only type: blue; bivalent-only type: red; appetitive-bivalent type: green). Colored and white areas depict the unpredicted and predicted reward-preferring neurons, respectively. **h**) Consistent sensitivity of reward predictability between the appetitive and bivalent blocks (*p* = 2.65^–24^, *ρ* = 0.59, Spearman’s correlation test). Each dot indicates a single lateral hypothalamus neuron. The same dot colors as in g were used to depict neuron types. (**i–j**) Preferred responses of reward predictability-coding neurons classified by their reward-predictability responses in the appetitive (**i**) or bivalent (**j**) blocks. The responses were indexed by the area under the curve value (AUC) during the test window (201–400 ms after US delivery) relative to the responses for the last 500 ms before timing cue (TC) onset (*p < 0.05/3, **p < 0.01/3, Wilcoxon signed-rank test with Bonferroni’s correction; **p < 0.01, Spearman’s correlation test). (**k–l**) Larger response modulation of the appetitive-bivalent (APP-BIV) type than that of the appetitive-only (APP-only) and bivalent-only (BIV-only) type (**k**) for the unpredicted reward-preferring neurons (*left*) and mean values during 201–400 ms after US onset (gray areas in k) for the positive (*left*) and negative (*right*) types (**l**). Normalized activity (*z*-score) in the free-reward trials was compared between the blocks for the unpredicted reward-preferring type (*left*), and that in the 100% trials was compared for the predicted reward-preferring type (*right*). **p < 0.05, one-way ANOVA and *post-hoc* Fisher’s least significant difference test. The same conventions are used as in Fig. 2.

To assess the neural valuation of outcomes depending on their predictability, we compared how well response modulation to rewards (201–400 ms) correlated with the predictability of reward delivery (100%, 50%, and free rewards). In the appetitive blocks, 35% (85/244) of the tested neurons exhibited a significant correlation (“reward predictability-coding” neurons). Approximately 47% (41/85) of them exhibited increased activity as the unpredictability of reward delivery increased (“unpredicted reward-preferring” neurons, 100% < 50% < free rewards; representative example, Fig. 3a; population, Fig. S2a–c), while the other 53% (44/85) exhibited increased activity as the predictability of reward delivery increased (“predicted reward-preferring” neurons, 100% > 50% > free rewards; representative example, Fig. 3d; population, Fig. S2d–f). In the bivalent blocks, 29% of the same population (71/244) was significantly modulated by reward predictability; 39 and 32 neurons were classified as unpredicted and predicted reward-preferring neurons, respectively. A subset of these neurons exhibited consistent reward-predictability coding between the appetitive and bivalent blocks (37/71; representative examples, Fig. 3a,b [unpredicted reward-preferring neuron] and 3d,e [predicted reward-preferring neuron]; population, Fig. 3g, *green*), but not in the aversive blocks (Fig. 3c,f). The other reward predictability-coding neurons encoded reward predictability in a context-dependent manner, either in the appetitive (48/85; appetitive-only type; Fig. 3g, *blue*) blocks or bivalent (34/71 in the bivalent blocks; Fig. 3g, *red*) blocks.

We also assessed the relationship of response sensitivity to reward predictability between the appetitive and bivalent blocks. A continuous cluster with a positive correlation was obtained (*p* < 0.01, Spearman’s correlation test; Fig. 3h), suggesting that reward-predictability coding was consistent between the appetitive and bivalent blocks at the population level. The neurons with significant correlations in the appetitive blocks (appetitive-only and appetitive-bivalent types) differentiated reward predictability more than those in the bivalent blocks (Fig. 3i, *left and center*), while the neurons with significant correlations in the bivalent blocks (bivalent-only and appetitive-bivalent types) exhibited more differential activity in the bivalent blocks compared to the appetitive blocks (Fig. 3j, *left and center*). These neurons did not differentiate the predictability of airpuff delivery (Fig. 3i,j, *right*). Further, the appetitive-bivalent type of the unpredicted reward-preferring neurons was largely involved in producing activity preference in the uncued-reward trials during the bivalent blocks (Fig. 3k,l). These results suggest that different subsets of neurons were recruited in a shared and context-dependent manner, which might contribute to the production of consistent reward-predictability signals with different outcome ranges.

We also quantified how many of the neurons responded consistently between the aversive and bivalent blocks depending on the predictability of airpuff delivery. A small but significant number of neurons (*n* = 22) were classified as punishment predictability-coding neurons (100% < 50% < free airpuff or 100% > 50% > free airpuff) in the aversive blocks. Approximately 36% (8/22) of them also showed significant correlations in the bivalent blocks, although the bivalent blocks could be interpretable with airpuff predictability. In contrast to the observation that all neurons consistently encoded reward predictability between the appetitive and bivalent blocks with the same correlation coefficient sign (Fig. 3h), two neurons (2/8) exhibited such consistency between the aversive and bivalent blocks (one for each type). These results suggest that the LH mainly encodes the valence of rewarding-unrewarding predictability, in addition to that of reward-punishment predictability.

### Uncertainty coding during the trace period with bidirectional responses

**Figure 4:**
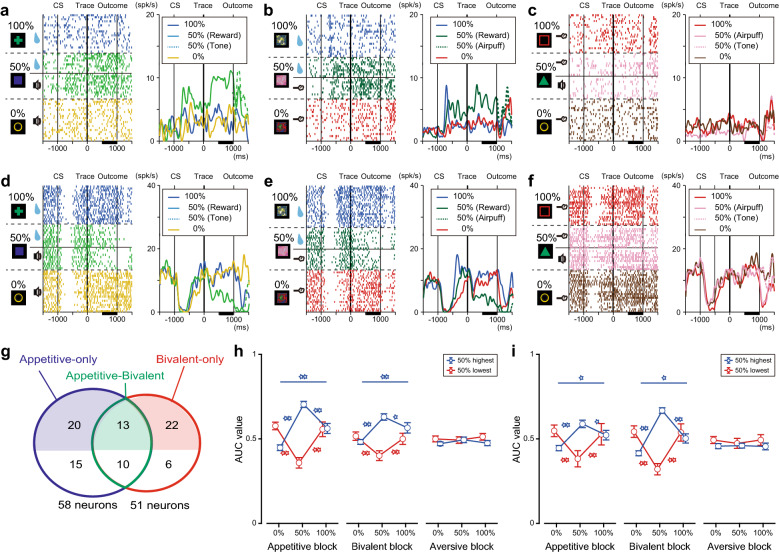
Responses of uncertainty-coding neurons in the lateral hypothalamus. (**a–f**) Representative responses of a 50%-highest neuron in the appetitive (**a**), bivalent (**b**), and aversive (**c**) blocks in the rasters (*left*) and histograms (*right*) and a 50%-lowest neuron in the appetitive (**d**), bivalent (**e**), and aversive (**f**) blocks. CS, conditioned stimulus. (**g**) Number of uncertainty-coding neurons in the appetitive and bivalent blocks (appetitive-only type: blue; bivalent-only type: red; appetitive-bivalent type: green). (**h–i**) Preferred response modulation of the 50%-highest and 50%-lowest neurons that were defined by their activity in the appetitive (**h**) and bivalent (**i**) blocks. Response modulation was assessed by the activity during the last 500 ms of the trace period relative to that during the last 500 ms before TC onset. *p < 0.05/3, **p < 0.01/3, Wilcoxon rank-sum test with Bonferroni’s correction.

Encoding reward uncertainty is one of the key features of the LH^28^, but it remains unknown how LH neurons respond in a bivalent context. The coding manner of reward (or punishment) uncertainty would generate a U-shape or inverted U-shape activity pattern as the reward (or punishment) probability increased in both appetitive (or aversive) and bivalent blocks^28^. To identify these activity patterns, neuronal activity was assessed during the last 500 ms of the trace period when the population activity was in parallel with the anticipatory licking and blinking behaviors. First, we confirmed the presence of such uncertainty-coding neurons in the appetitive and bivalent blocks. In the appetitive blocks, 24% of neurons (58/244) exhibited the highest (“50%-highest” type, 33/58; representative example, Fig. 4a; population, Fig. S3a–c; inverted U-shape-like activity) or lowest (“50%-lowest” type, 25/58; representative example, Fig. 4d; population, Fig. S3d–f; U-shape-like activity) activity in the 50% trials during the last 500 ms of the trace period (“uncertainty-coding” neurons). A subset of uncertainty-coding neurons showed consistent activity between the appetitive and bivalent blocks (appetitive-bivalent type, *n* = 23; representative examples, Fig. 4a,b and d,e), but not in the aversive blocks (Fig. 4c,f). Compared to the uncertainty-coding neurons defined in the appetitive blocks, a smaller number of neurons (51/244) encoded uncertainty in the bivalent blocks (50%-highest type: 35/51, 50%-lowest type: 16/51). We also found that different subsets of these uncertainty-coding neurons were recruited in a context-dependent manner, either in the appetitive (appetitive-only type, *n* = 35; Fig. 4g, *blue*) blocks only or bivalent (bivalent-only type, *n* = 28; Fig. 4g, *red*) blocks only. These context-dependent uncertainty coding-neurons were larger subpopulations than the shared uncertainty-coding neurons (appetitive-bivalent type, *n* = 23; Fig. 4g, *green*). Unlike the coding of CS values and US predictability, these uncertainty-coding neurons selectively contributed to the production of similar response modulation in the appetitive (Fig. 4h) and bivalent (Fig. 4i) blocks.

In the aversive blocks, 5% (12/244) of neurons encoded the punishment uncertainty signals (50%-highest type, 5/12; 50%-lowest type, 7/12). This was detected statistically by chance. Thus, the neuronal activity in the LH during the trace period primarily encodes rewarding-unrewarding uncertainty.

### Consistent signals between the appetitive and bivalent blocks

**Figure 5:**
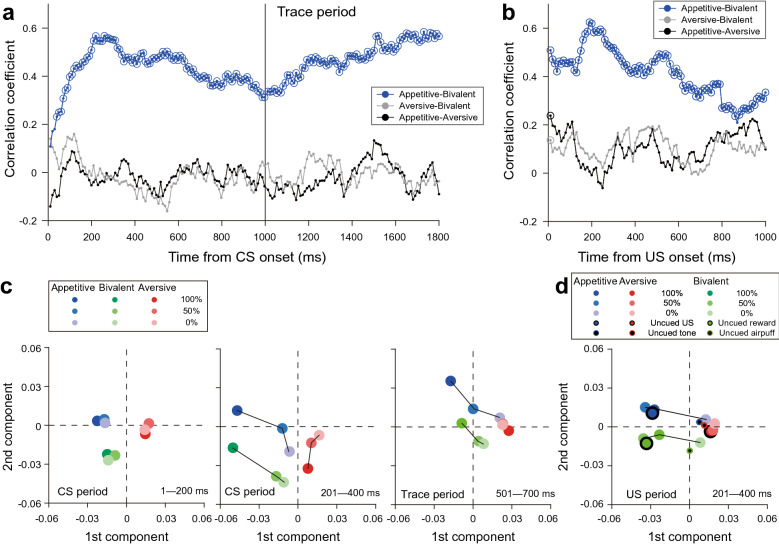
Consistent activity throughout a trial between the appetitive and bivalent blocks. (**a, b**) Consistent conditioned stimulus (CS) value-coding during CS and trace periods (**a**) and outcome predictability coding (**b**). Each dot represents the correlation coefficient between the appetitive and bivalent blocks (blue), between the aversive and bivalent blocks (gray), and between the appetitive and aversive blocks (black) at the population level. Spearman’s correlation test was applied in each test window with a 200-ms duration moving in 10-ms steps. The circles represent statistical significance (*p* < 0.05). Dots and circles were aligned at the initial time point of the test window. All neurons were used for the calculation. US, unconditioned stimulus. (**c**) Clear differentiation among trial types and blocks in the principal component feature space (1–200 ms after CS onset, *left*; 201–400 ms after CS onset, *center*; 501–1,000 ms after trace onset, *right*). For feature-space production, the first and second components of principal component analysis (PCA) were used. Data used in PCA were the two-dimension matrix of all neurons (*n* = 244) × averaged firing rates of 540 bins in total in the 100% and 0% trial types during the appetitive-bivalent, and aversive blocks (90-time bins × 2 trial types × 3 blocks). Each bin had a 100-ms duration and moved from CS onset to the end of the trace without overlap for each block. Black lines linking cue-trial types in the same block were added for better recognition. (**d**) Valid representation of uncued and cued outcome values in the principal component feature space. Despite using only the CS and trace activity in the 100% and 0% trials, the feature space well represents the values of the uncued outcomes: large blue, green, and red circles with the black edge depict free reward in the appetitive block, free reward in the bivalent block, and free airpuff in the aversive blocks, respectively; small black dots with the blue, green, and red edges illustrate free tone in the appetitive block, free airpuff in the bivalent block, and free tone in the aversive blocks, respectively.

To assess how long the similar responses between the appetitive and bivalent blocks were preserved after CS onset at the population level, we measured the sensitivity of all individual neurons to the graded CS values (i.e., correlation coefficient) during the CS and trace periods using a sliding window technique (200-ms duration with a 10-ms step) and calculated the correlation coefficients between the blocks at the population level. We found a significantly positive correlation between the appetitive and bivalent blocks until the end of the trace period (Fig. 5a, *blue*), but not between the aversive and bivalent blocks (Fig. 5a, *gray*) or between the appetitive and aversive blocks (Fig. 5a, *black*). After outcome delivery, we also measured the independent sensitivity of each neuron to US predictability. Consistent significant correlations in US predictability were obtained only between the appetitive and bivalent blocks (Fig. 5b). These results suggest that the LH encodes rewarding-unrewarding valence signals consistently throughout a trial.

Such temporal preservation was verified by principal component analysis (PCA; Fig. 5c). PCA was applied to a data matrix consisting of the mean activity of each neuron in the 100% and 0% trials during the CS and trace periods in all blocks and transformed values in the feature space of the first and second principal components. In this space, the values among the three blocks were differentiated immediately after the CSs appeared (Fig. 5c, *left*). Consistent with the results of the CS value-coding neurons in regression analysis, the trial types in all blocks were also clearly separated after 200 ms (Fig. 5c, *center*). These clear differentiations among the blocks and probabilities in each block indicate that the LH can encode rewarding-unrewarding valence signals (appetitive vs. bivalent blocks) and reward-punishment valence signals (appetitive [bivalent] vs. aversive blocks) at the population level. Moreover, such separation in the bivalent blocks was paralleled by that in the appetitive blocks, which was preserved at even 500 ms after trace-period onset (Fig. 5c, *right*), while the separation of punishment probabilities in the aversive blocks was diminished. Notably, this feature space also well represented the responses to outcomes in the appetitive and aversive blocks, despite using only the CSs and trace responses in the 100% and 0% trials for feature-space construction (Fig. 5d). This suggests a consistent representation of the rewarding-unrewarding valence signals throughout a trial. Although neuronal activity was not recorded simultaneously, PCA revealed the predominant processing of rewarding-unrewarding valence signals and context signals throughout a trial, in addition to reward-punishment valence signals.

### Baseline activity and TC activity

As the LH is thought to process motivational and arousal components, different types of neuronal activity can be observed at the context level in the baseline and TC activity^28^. A comparison of these types of activity (baseline activity: last 1,000 ms; TC activity: 101–500 ms) among the three blocks revealed that a sufficient number of neurons exhibited differential response modulation among the blocks at the cellular level (baseline activity: 70% [171/244]; TC activity: 54% [131/244]; *p* < 0.05, one-way ANOVA). However, there was no significant activity at the population level (baseline activity: *F*_[2,731]_ = 0.04, *p* = 0.96; TC activity: *F*_[2,731]_ = 0.25, *p* = 0.78; one-way ANOVA), and a comparable number of neurons with significant differential activity between two different blocks was obtained (baseline activity: *n* = 102 [appetitive vs. aversive blocks], *n* = 110 [appetitive vs. bivalent blocks], *n* = 114 [aversive vs. bivalent blocks]; TC activity: *n* = 72 [appetitive vs. aversive blocks], *n* = 74 [appetitive vs. bivalent blocks], *n* = 84 [aversive vs. bivalent blocks]; *p* < 0.05/3, Welch’s *t*-test with Bonferroni’s correction). These results suggest that the LH reflects context information, presumably including motivational and arousal signals, at the cellular level, but not at the population level. These observations also indicate that a variety of neuron types in the LH may be recruited for the consistent baseline and TC responses among the blocks.

## Discussion

The present study assessed how behavioral and neuronal responses in the primate LH were modulated under a bivalent situation. Behaviorally, the animals sustained reward valuation and attenuated punishment valuation in the bivalent blocks against the possibility that they could have a lower reward valuation in the bivalent blocks than in the appetitive blocks. The mean population activity in the LH was correlated with these approaching and avoiding behaviors. Further, we found that the predominant coding manner of “rewarding-unrewarding” valence signals was related to prediction such as expectation, predictability, and uncertainty with bidirectional responses. These valence signals were preserved consistently throughout a trial at the population level. PCA revealed that the LH encoded information on context and valence among different contexts. Thus, in addition to the notion of “reward-punishment” valence coding in the LH, our data suggest predominant “rewarding-unrewarding” valence coding for motivational impact on graded behaviors in bivalent contexts.

The positive type CS value-coding neurons presumably reflected reward expectancy. The 50%-highest and unpredicted reward-preferring neurons were associated with reward uncertainty and prediction-error signals, respectively. However, the negative type CS value-coding neurons in the bivalent blocks may represent the punishment and unrewarding probability or merely flip the activity of the positive type CS value-coding neurons. Correspondingly, it remains unclear whether the predicted reward-preferring neurons in the bivalent blocks encoded reward predictability or the unpredictability of punishment. For the uncertainty-coding neurons, it is also ambiguous whether they encoded the reward uncertainty or the certainty of punishment in the bivalent blocks. Therefore, it is important to clarify how these neuron types process punishment prediction signals. By comparing neuronal responses to prediction-related events among the three blocks, we confirmed that the LH predominantly processes opposing valences of fine rewarding-unrewarding valence signals, i.e., how likely and unlikely rewards will be delivered, how uncertain and certain animals are that they will obtain rewards, and how well the received rewards are unpredicted or predicted, in addition to reward-punishment valence signals.

The LH has been proposed to process positive (i.e., reward) and negative (i.e., punishment) motivational signals^28,32,33^. The positive motivational signals are plausibly mediated by the circuit of the LH with the ventral tegmental area (VTA). In particular, dopaminergic neurons in the VTA respond to cues predicting reward and unpredictable reward delivery, indicating that they carry reward prediction (error) signals^34^. In the present study, the LH exhibited a similar activity pattern such as the positive-type CS-value coding neurons and the unpredicted reward-preferring type. This neuronal activity may contribute to producing or reflecting dopaminergic activity in the VTA^32,35^. For the negative motivational signals, the LH-VTA circuit plays an essential role. Further, the interactions of the LH with the lateral habenula^36^, globus pallidus^37^, and amygdala^38^ may mediate aversive signals and negative motivational signals. Similarly to lateral habenula neurons^39^, the subpopulations of LH neurons responded to punishing and unrewarding conditioning events with or without dependency on punishment probability. These neurons might be involved in the graded anticipatory blinking. Recruiting these neurons in concert with other types of neurons in the LH and those in the VTA, lateral habenula, globus pallidus, and amygdala can facilitate the processing of a wide range of motivational signals in the LH for adaptive approaching and avoiding behaviors.

Indeed, the feature space of principal components well captured such positive and negative motivational signals, including context information (Fig. 5b,c). Despite the lack of the simultaneous recording of these neurons, these findings suggest that the LH processes signals ranging from rewards to punishments with a high sensitivity to rewards by discriminating its probabilities. This led to the apparently different notions that the LH encodes rewarding-unrewarding and reward-punishment valence signals. These signals may provide adaptive motivational signals against different contexts. Such a manner of coding might be beneficial for shaping robust motivational signals in different contexts, particularly under equivocal situations, by evaluating the valence of good and bad outcomes via attenuating negative valence or regarding negative valence as neutral valence (i.e., rectification) for downstream neurons^40^.

A caveat is that such neuronal activity might merely reflect arousal or the salience of task events. However, this cannot fully explain our findings, in particular, the observation that a smaller number of neurons in the LH carried punishment probability or predictability information in the aversive blocks than those for reward probability or predictability information. If the neuronal responses to rewarding and punishing events had reflected arousal or salience, more neurons would have responded in the bivalent blocks than in the appetitive and aversive blocks, which was not the case at least at the population level. These findings support the notion that the LH encodes motivational valence. Another caveat is that we used airpuffs as a punishment but did not examine behaviors and neuronal activity using different intensities of airpuffs or procedures such as tail pinch or electric foot shock frequently conducted in rodent studies. These might result in a different coding manner for aversive prediction signals in the LH.

The bivalent blocks were always conducted after the appetitive and aversive blocks in our procedure. Recently, an optogenetics study revealed that gamma-aminobutyric acid neurons in the LH are critically involved in aversive learning after reward learning^33^. That study suggested that past experience shapes the neural circuits recruited for future valence learning, including appetitive, aversive, and conceivably bivalent situations. This effect may be mediated by a different mechanism from trial-based hysteresis such as the impact of prior outcomes on subsequent responses (Supplementary information). Further studies are necessary to elucidate this mechanism by manipulating context order (i.e., past experience) and memory effects before, during, and after learning.

Moreover, little is known about how these prediction signals are used by downstream neurons to integrate specific internal demands and link them to motivational behaviors^7,27^, especially in multivalent and dynamic contexts such as social situations^41,42^. Advanced new tools such as optogenetics and designer drugs will be useful for associating prediction signals with electrophysiological properties (Table S1) and specific contributable circuits for these behaviors. For example, the 50%-highest neurons had different electrophysiological aspects compared to the other types. This type might receive reward certainty or uncertainty information from upstream neural circuits, including the lateral and medial prefrontal cortex^43,44^ and septum^44,45^. Thus, our findings are a foundation to reveal what neuronal information in the LH drives adaptive approaching and avoiding behavior in bivalent contexts.

## Method

### General

The procedures, except the bivalent blocks, are described in detail elsewhere^28^. Briefly, we recorded single-unit activity from the LH in three hemispheres of two male cynomolgus monkeys (*Macaca fascicularis*; monkey F, 5 kg, left hemisphere; monkey S, 5 kg, both hemispheres). Water intake was mildly restricted, and they were therefore thirsty during the experiments. All experimental procedures were performed in accordance with the National Institutes of Health Guidelines for the Care and Use of Laboratory Animals and approved by the Institutional Animal Care and Use Committee at Kansai Medical University. This study is reported in accordance with ARRIVE guidelines^46^.

The experimental setting was the same as described previously (Fig. 1a)^28^. Visual stimuli were rear-projected onto a fronto-parallel screen that was placed 68 cm in front of the monkey at eye level by a projector (ELP-505; EPSON, Nagano, Japan). Licking was monitored by a vibration sensor attached to a reward spout (AE-9922; NF Corporation, Kanagawa, Japan), and eye position was collected by an infrared video camera set below the screen at a time resolution of 360 Hz with a spatial resolution of 0.1° (EYE-TRAC6; ASL, Bedford, MA, USA). Neuronal signals were amplified and filtered (50 or 100 Hz–10 kHz; MEG-5100; Nihon Kohden, Tokyo, Japan). A template-matching spike discriminator was used to isolate single-unit activity at a time resolution of 50 or 40 kHz for waveform matching and spike sampling^47^ (Alpha-Omega, Nazareth, Israel; or OmniPlex system; Plexon, Inc., Dallas, TX, USA). Isolated spike timing, licking behavior, and eye positions were eventually sampled at 1 kHz. A data acquisition system (Tempo; Reflective Computing, Olympia, WA, USA) controlled the aspects of stimuli presentation, monitoring of eye movements and neuronal activity, and reward delivery.

### Pavlovian procedure

The animals were conditioned with a Pavlovian trace procedure in three distinct blocks of trials: appetitive, aversive, and bivalent blocks (Fig. 1c). Each block consisted of cued and uncued trials (Fig. 1b). In the cued trials in each block, three different visual images (10° of the visual field) were used as CSs. Each CS was associated with the delivery of a US consisting of a water or apple juice reward (0.1 mL) in the appetitive blocks or an airpuff (0.01–0.05 MPa) as a punishment in the aversive blocks with a probability (100%, 50%, or 0%). In the bivalent blocks, both were used. A trial started with the presentation of a TC (white dot, 12° of the visual field) for 1.2 s at the center of the screen to obtain the animal’s attention. After the disappearance of the TC, one of three CSs was presented for 1 s, followed by a 1-s trace period with a black screen. The outcome was then delivered for 100 ms. When the USs (i.e., rewards or airpuffs) were not delivered in the 50% or 0% trials in the appetitive and aversive blocks, a tone was delivered instead. During the bivalent blocks, an airpuff was delivered instead of the tone. Thus, the 100%, 50%, and 0% trial types in the bivalent blocks indicate the probability of reward delivery. In the uncued trials, a reward (free reward) or a tone (free tone) alone was delivered during the appetitive blocks, and an airpuff (free airpuff) or a tone alone was delivered during the aversive blocks. In the bivalent blocks, a reward or an airpuff alone was delivered. The free outcome was delivered at the time corresponding to that of outcome delivery in the cued trials. A block comprised 80 trials with a fixed proportion of trial types (each CS, 20 trials; free reward, free tone, or free airpuff, 10 trials). The cued and non-cued trials were presented pseudorandomly with an inter-trial interval of 3–5 s. The appetitive and aversive blocks were conducted in a roughly random order on each experimental day^28^, while the bivalent blocks were always conducted after these blocks on the same day.

### Identification of the LH

The recording chambers were installed over the frontoparietal cortices, laterally angled at 20° (monkey F) or 35° (monkey S) to access the LH. Recording sites were confirmed by overlaying penetration record maps on magnetic resonance images (0.3 T, AIRIS; Hitachi, Tokyo, Japan; Fig. 1d). The LH is located at 1 mm anterior and 7 mm posterior to the anterior commissure, ventrally adjacent to the internal capsule, globus pallidus, and zona incerta, and medially adjacent to the substantia nigra pars reticulata^48^. To identify the LH, we referred to these regions as useful landmarks^28^. Electrolytic microlesions were made in the recording sites in the LH of monkey S and verified that the neurons were recorded from the LH^28^. To assess whether the encountered neurons were located in the LH, we also examined neuronal responses to the sight of food pieces and the unexpected delivery of juice or an airpuff before the recordings^28,29,49–52^.

### Analysis of behavioral data

Anticipatory licking and blinking were analyzed to assess the animals’ valuation of CSs as behavioral measures of reward expectancy and punishment avoidance, respectively. Licking and blinking data were normalized as *X*/*Max*, where *X* was the mean frequency during the 250 ms before outcome delivery in a particular condition, and *Max* was the maximum frequency in each recording session. Most anticipatory licking and blinking were observed in the analyzed period. Statistical analysis was performed using the Wilcoxon signed-rank test with Bonferroni’s correction (*p* < 0.05/3; Fig. 1e). We used two-way ANOVA with probabilities and contexts as factors to detect differences in blinking frequency between the aversive and bivalent blocks.

### Analysis of neuronal activity

We examined the responses of 244 neurons (127 in monkey F, 117 in monkey S) in the LH during three different outcome contexts among the task-related neurons (*n* = 308) identified in a previous study^28^. The task-related neurons were responsive to at least one of the conditioning events in either the appetitive or aversive block or both^28^ (one-way repeated measures ANOVA, *p* < 0.01). Since the 244 neurons analyzed in the current study were a subset of task-related neurons in a previous study^28^, they had the electrophysiological properties of neurons in the LH. We combined neuronal data in two monkeys as in our previous study^28^.

For population activity comparison among the three blocks, the activity during the CS and trace periods was normalized (z-score) using the activity for the last 500 ms before TC onset in individual neurons for each block. The population activity of 244 neurons was compared among the three blocks independently in each window (200-ms duration with a 50-ms step) in which one-way ANOVA and *post-hoc* Tukey–Kramer test were applied to detect significantly lower activity in the aversive blocks compared to the appetitive and bivalent blocks (*p* < 0.05).

Linear regression analysis was performed to determine whether neuronal activity reflected the probability, uncertainty, and predictability of outcome delivery^28^. To analyze the CS values, how well the CS responses (201–400 ms from CS onset) were graded depending on the values associated with US probabilities (CS values) was determined in each neuron. Predictability of outcome delivery was estimated by assessing how well the US responses (201–400 ms) were modulated in each neuron by the predictability of US delivery (i.e., 100%, 50%, and free USs). Outcome uncertainty was evaluated to analyze the association between the last 500 ms of activity during the trace period and the degree of US uncertainty (100% and 0% trials vs. 50% trials). These time windows captured the primary response modulation of neurons in the LH. To examine the temporal relationship of activity between two blocks, we applied regression analysis to the sensitivity to CS values (i.e., correlation coefficient; Fig. 5a) and outcome predictability (Fig. 5b) between the bivalent and appetitive blocks, between the bivalent and aversive blocks, and between the appetitive and aversive blocks for all neurons in each analyzed window (200-ms duration and 10-ms steps).

In PCA, we used two-dimension matrix data (neurons [*n* = 244] × averaged firing rates in the 100% and 0% trial types in the appetitive-bivalent, and aversive blocks, which were concatenated [540 bins in total: 90-time windows × 2 trial types × 3 blocks]). Each time bin had a 100-ms duration and moved in 100-ms steps from CS onset to the end of the trace. The first and second components were used to construct the feature space and transform the values of the CS (Fig. 5c, *left and center*), Trace (Fig. 5c, *right*), and US (Fig. 5d) responses.

### Analysis of the electrophysiological characteristics of neurons

We analyzed the baseline activity and spike-wave shape of the sampled neurons to characterize their physiological properties. We calculated the mean spike duration from the first sharp trough to the peak of the second long-duration positive deflection measured in the whole appetitive block for each neuron. All measures were compared between each neuron type and neurons without statistical significance (*n.s.* type) using the Wilcoxon rank-sum test with Bonferroni’s correction (*p* < 0.05/6).

## Supplementary Information

Supplementary Information.

## Data Availability

All data needed to evaluate the conclusions in the paper are presented in the paper.
