# Supplementary material for: Rewarding-unrewarding prediction signals under a bivalent context in the primate lateral hypothalamus
Source: Sci Rep. 2023 Apr 12;13:5926. doi: 10.1038/s41598-023-33026-0 (PMC10097697; doi:10.1038/s41598-023-33026-0)
Supplement: Supplementary file 1 — Supplementary Information. [file 41598_2023_33026_MOESM1_ESM.docx]

**Supplementary Information and Figures**

# **Rewarding-unrewarding prediction signals under a bivalent context in the primate lateral hypothalamus**

Atsushi Noritake and Kae Nakamura

***Electrophysiological properties***

The LH contains a variety of neurons with different neurochemical profiles^1–5^. To identify the electrophysiological properties of the recorded neurons, we examined how they differed in baseline activity and spike shapes during the baseline period (**Table S1**). The neurons with the 50%-highest responses in the appetitive and bivalent blocks were characterized by significantly lower baseline activity and shorter spike widths than those without significance for uncertainty (i.e., *n.s.* neurons). In addition, the baseline activity of the CS value-encoding neurons in the bivalent blocks was significantly lower than that of those without significance. However, there was no significant difference in baseline activity and spike width in the other types of neurons except for the baseline activity of the negative CS value-coding neurons. There was also no significant difference in the distribution of neuron types in the recorded location^6^. These data suggest that the neuron types were intermingled in the LH, consistent with previous studies^6,7^.

***Hysteresis effect***

Previous studies suggest that the lateral hypothalamus is also profoundly involved in memory functions^6,8–13^. To study how prior events influenced neurons in the lateral hypothalamus in the bivalent blocks, we examined the impact of the outcome in the previous trial on baseline and timing cue (TC) activities in the current trial. For this analysis, we applied regression testing to assess the impact of the outcome in the previous trial on baseline activity (1–1,000 ms before TC onset) and TC activity (101–500 ms after TC onset) in the current trial for individual neurons in each block. The outcome of the previous trial exerted a significant influence on the baseline activity of more than 10% of neurons in the current trial during the appetitive (27/244) and bivalent (29/244) blocks, whereas this was observed in only seven neurons in the aversive block. There was a significant correlation between the TC activity of the current trial and the outcome of the previous trial in approximately 18% of neurons in the appetitive (44/244) and bivalent (45/244) blocks, but only 11% of neurons showed such a correlation in the aversive block (26/244). Hence, hysteresis effects were relatively larger in the appetitive and bivalent blocks than in the aversive blocks. These results suggest that outcomes with positive valence, more than ones with negative valence, influence baseline and TC activity in the subsequent trials.

**References**

1. Patricia Bonnavion, Laura Mickelsen, Fujita, A., de Lecea, L. & Jackson, A. C. Hubs and spokes of the lateral hypothalamus: cell types, circuits and behavior. *J Physiol.* **594**, 6443–6462 (2016).

2. González, J. A., Iordanidou, P., Strom, M., Adamantidis, A. & Burdakov, D. Awake dynamics and brain-wide direct inputs of hypothalamic MCH and orexin networks. *Nat. Commun.* **7**, 11395 (2016).

3. Sharpe, M. J. *et al.* Lateral hypothalamic GABAergic neurons encode reward predictions that are relayed to the ventral tegmental area to regulate learning. *Curr. Biol.* **27**, 2089-2100.e5 (2017).

4. Marino, R. A. M. *et al.* Control of food approach and eating by a GABAergic projection from lateral hypothalamus to dorsal pons. *Proc. Natl. Acad. Sci. U. S. A.* **117**, 8611–8615 (2020).

5. Hassani, O. K., Henny, P., Lee, M. G. & Jones, B. E. GABAergic neurons intermingled with orexin and MCH neurons in the lateral hypothalamus discharge maximally during sleep. *Eur. J. Neurosci.* **32**, 448–457 (2010).

6. Noritake, A. & Nakamura, K. Encoding prediction signals during appetitive and aversive Pavlovian conditioning in the primate lateral hypothalamus. *J. Neurophysiol.* **121**, 396–417 (2019).

7. Sakurai, T. *et al.* Input of orexin/hypocretin neurons revealed by a genetically encoded tracer in mice. *Neuron* **46**, 297–308 (2005).

8. Hsu, T. M., Suarez, A. N. & Kanoski, S. E. Ghrelin: A link between memory and ingestive behavior. *Physiol. Behav.* **162**, 10–17 (2016).

9. Burdakov, D. & Peleg-Raibstein, D. The hypothalamus as a primary coordinator of memory updating. *Physiol. Behav.* **223**, 112988 (2020).

10. Han, D., Han, F., Shi, Y., Zheng, S. & Wen, L. Mechanisms of memory impairment induced by orexin-A via orexin 1 and orexin 2 receptors in post-traumatic stress disorder rats. *Neuroscience* **432**, 126–136 (2020).

11. Petrovich, G. D. Forebrain circuits and control of feeding by learned cues. *Neurobiol. Learn. Mem.* **95**, 152–158 (2011).

12. Devarakonda, K. & Kenny, P. J. Energy balance: lateral hypothalamus hoards food memories. *Curr. Biol.* **27**, R803–R805 (2017).

13. Sharpe, M. J., Batchelor, H. M., Mueller, L. E., Gardner, M. P. H. & Schoenbaum, G. Past experience shapes the neural circuits recruited for future learning. *Nat. Neurosci.* **24**, 391–400 (2021).

**Table S1**. Medians and interquartile ranges (in parentheses) of the baseline activity for the conditioned stimulus (CS) value-coding neurons (*top*), predictability-coding neurons (*middle*), and uncertainty-coding neurons (*bottom*). **p* < 0.05/6, ***p* < 0.01/6, significant difference in baseline activity between the targeted neurons and neurons without significance (*n.s.* neurons) in each block (Wilcoxon rank-sum test with Bonferroni’s correction). These statistical tests (each type vs. *n.s.* type) were performed independently in each block.

**
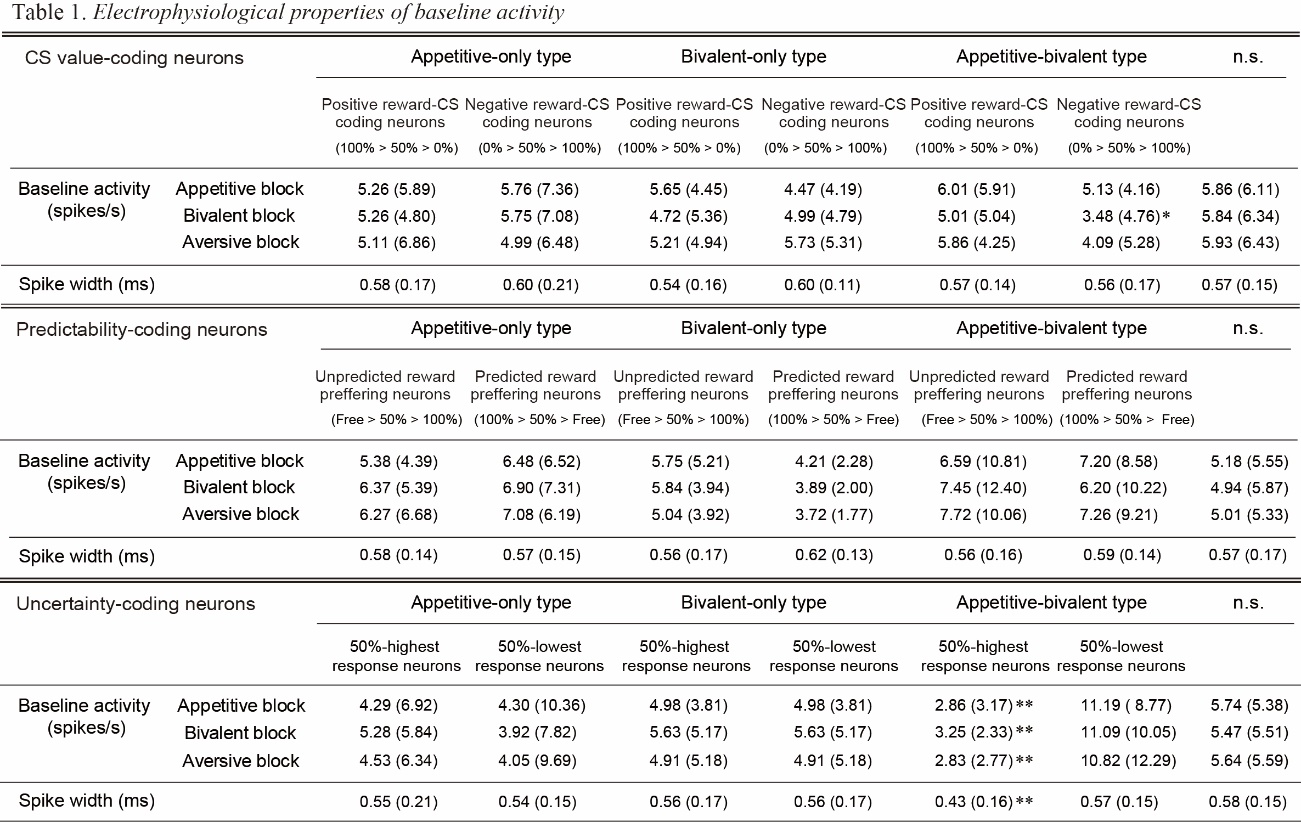
**

**Supplementary figures**

**
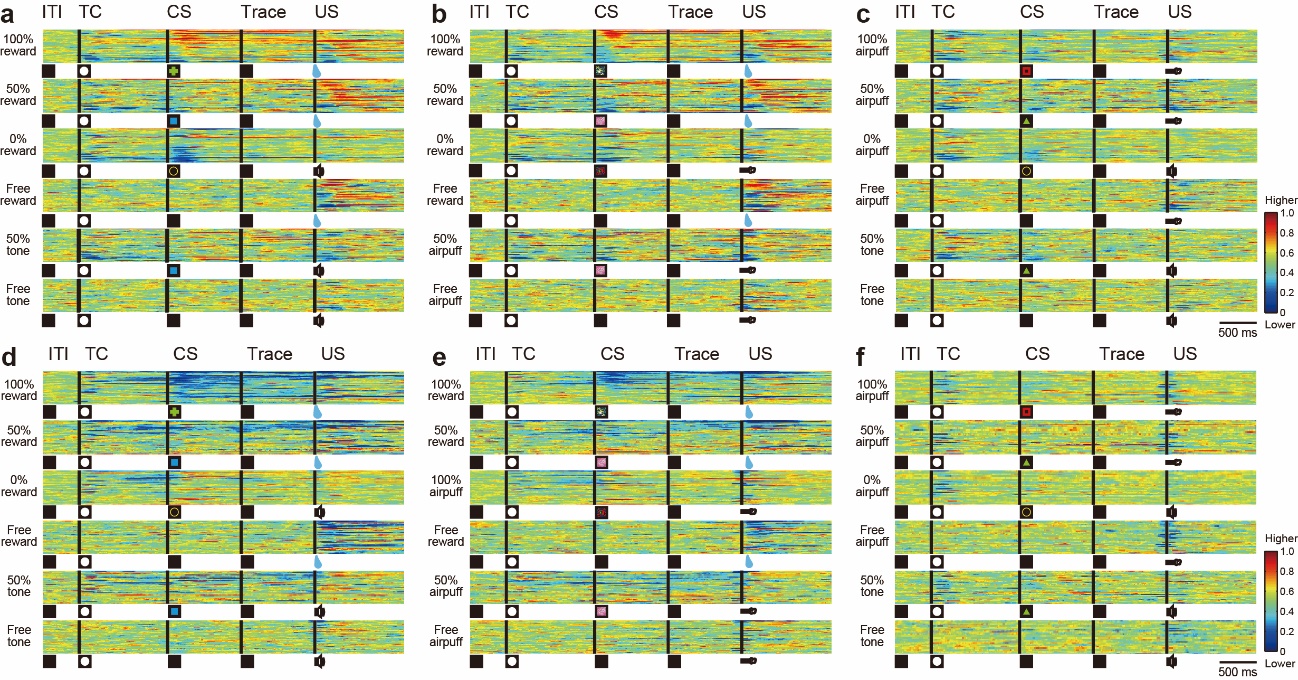
**

**Fig. S1**

Population activity of the positive (**a–c**) and negative (**d–f**) types of CS value-coding neurons in the appetitive (**a,d**), bivalent (**b**,**e**), and aversive (**c**,**f**) blocks. The activity of each neuron is represented as a row. Neurons are sorted according to the strength and duration of their responses to the 100% reward CS in the appetitive blocks separately for the positive and negative types. The same order was applied to all blocks for each type. The color of each pixel depicts the value of the area under the receiver operating characteristic curves of the firing rate during a test window relative to that during the period at 500 ms before timing cue (TC) onset. Pixels are centered on the midpoint (mean time) of the corresponding test windows. Each window has a 200-ms duration and moves in 10-ms steps. Note that CS value-coding neurons that were classified by their CS responses in the appetitive blocks are shown.

**
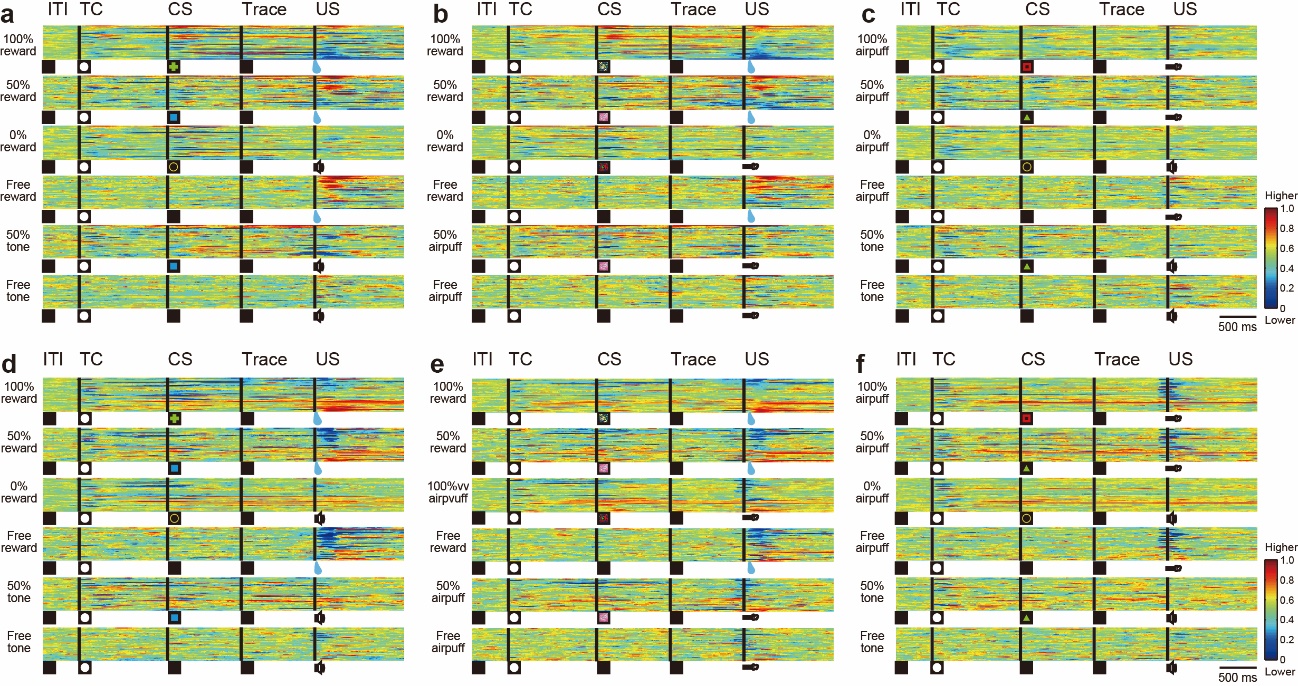
**

**Fig. S2**

Population activity of the unpredicted (**a–c**) and predicted (**d–f**) reward-preferring types of reward-predictability coding neurons in the appetitive (**a,d**), bivalent (**b**,**e**), and aversive (**c**,**f**) blocks. Neurons are sorted according to the strength and duration of their responses to the free reward in the appetitive blocks separately for the unpredicted and predicted reward-preferring types. The reward predictability-coding neurons that were classified by their responses to reward delivery in the appetitive blocks are shown. The same order was applied to all blocks for each type. The same format as in Fig. S1.

**
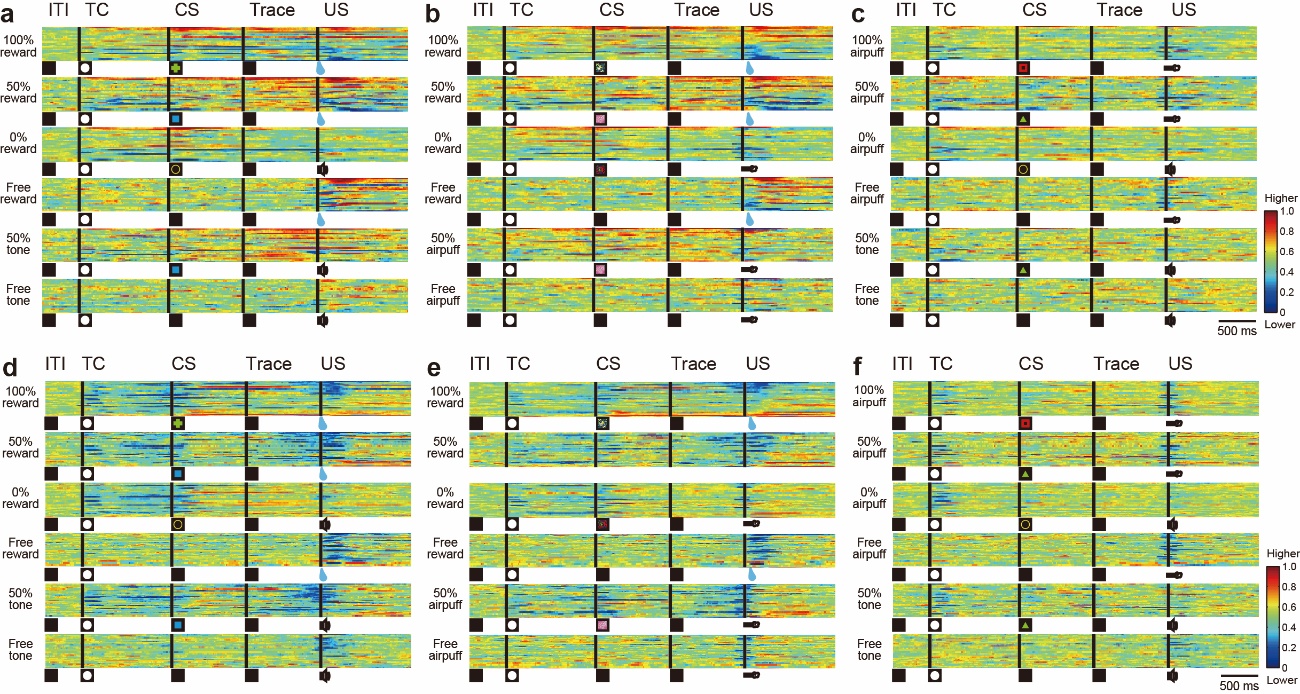
**

**Fig. S3**

Population activity of the 50%-highest (**a–c**) and 50%-lowest (**d–f**) types of reward-uncertainty coding neurons in the appetitive (**a,d**), bivalent (**b**,**e**), and aversive (**c**,**f**) blocks. Neurons are sorted according to the strength and duration of their responses to the 50% reward during the trace period in the appetitive blocks separately for the unpredicted and predicted reward-preferring types. The reward uncertainty-coding neurons that were classified by their responses during the trace period in the appetitive blocks are shown. The same conventions are used as in Figs. S1 and S2.
